# An intronic single-nucleotide polymorphism (rs13217795) in *FOXO3* is associated with asthma and allergic rhinitis: a case–case–control study

**DOI:** 10.1186/s12881-017-0494-4

**Published:** 2017-11-15

**Authors:** Justin Z. Amarin, Randa G. Naffa, Haya H. Suradi, Yousof M. Alsaket, Nathir M. Obeidat, Tareq M. Mahafza, Malek A. Zihlif

**Affiliations:** 1 School of Medicine, The University of Jordan, Amman, Jordan; 2 Molecular Biology Research Laboratory, School of Medicine, The University of Jordan, Amman, Jordan; 3 School of Dentistry, The University of Jordan, Amman, Jordan; 4 Department of Internal Medicine, School of Medicine, The University of Jordan, Amman, Jordan; 5 Department of Special Surgery, School of Medicine, The University of Jordan, Amman, Jordan; 6 Department of Pharmacology, School of Medicine, The University of Jordan, Queen Rania Al-Abdullah Street, Amman, 11942 Jordan

**Keywords:** FOXO3, Asthma, Allergic rhinitis, Single-nucleotide polymorphism, rs13217795

## Abstract

**Background:**

Asthma and allergic rhinitis are respiratory diseases with a significant global burden. Forkhead box O3 (*FOXO3*) is a gene involved in the etiology of a number of respiratory diseases. The objective of this study is to assess the association of rs13217795, an intronic *FOXO3* single-nucleotide polymorphism, with asthma and allergic rhinitis.

**Methods:**

In this case–case–control genetic association study, genotyping was conducted using the PCR–RFLP method. Genotype-based associations were investigated under the general, recessive, and dominant models of disease penetrance using binomial logistic regression; and, allele-based associations were tested using Pearson’s chi-squared test.

**Results:**

The final study population consisted of 94 controls, 124 asthmatics, and 110 allergic rhinitis patients. The general and recessive models of disease penetrance were statistically significant for both case–control comparisons. Under the general model, the odds of the asthma phenotype were 1.46 (0.64 to 3.34) and 3.42 (1.37 to 8.57) times higher in heterozygotes and derived allele homozygotes, respectively, compared to ancestral allele homozygotes. The corresponding odds ratios for the allergic rhinitis phenotype were 1.05 (0.46 to 2.40) and 2.35 (0.96 to 5.73), respectively. The dominant model of disease penetrance was not statistically significant. The minor allele in all study groups was the ancestral allele, with a frequency of 0.49 in controls. There was no deviation from Hardy–Weinberg equilibrium in controls. Both case–control allele-based associations were statistically significant.

**Conclusions:**

Herein we present the first report of the association between rs13217795 and allergic rhinitis, and the first independent verification of the association between rs13217795 and asthma. Marker selection in future genetic association studies of asthma and allergic rhinitis should include functional polymorphisms in linkage disequilibrium with rs13217795.

**Electronic supplementary material:**

The online version of this article (10.1186/s12881-017-0494-4) contains supplementary material, which is available to authorized users.

## Background

Asthma is a chronic respiratory disease characterized by airway inflammation and remodeling [1]. The number of asthmatics worldwide is estimated to be 235 million, and the global burden of this disease is projected to rise [2]. Asthma is a polygenic, multifactorial disorder; more than a hundred susceptibility genes have been identified [3]. The complex risk architecture of asthma is typified by involvement of both genetic and environmental factors [4]. Indeed, asthma is not a singular disease entity. The novel concept of asthma endotypes emerges from this understanding [5]. The clinical delineation of asthma subtypes will further enable benchside-to-bedside translation of genetic studies.

Allergic rhinitis, an IgE-mediated disease of the nasal mucosa, is the most common form of noninfectious rhinitis. The prevalence of allergic rhinitis in the global population is at least twice that of asthma [6]. Allergic rhinitis and asthma are interrelated inflammatory disorders. The former also involves mucosal remodeling, albeit to a lesser extent [7]. It has been postulated that asthma and allergic rhinitis are analogous inflammatory disorders of a continuous airway under the “integrated airway” hypothesis [8].

Forkhead box O3 (*FOXO3*) is a protein-coding gene on chromosome 6q21. The product, forkhead box protein O3 (FOXO3), is a member of the forkhead family of transcription factors. These transcription factors play a key role in immune homeostasis; as such, loss-of-function variants may be associated with chronic inflammatory processes [9]. *FOXO3* variation has been implicated—through a myriad of mechanisms—in the etiology of idiopathic pulmonary fibrosis, non-small cell lung cancer, chronic obstructive pulmonary disease, and bronchiolitis [10–15].

A recent human genome assembly by the Genome Reference Consortium (GRCh38.p7) identified 6499 (5372 intron) single-nucleotide variants in *FOXO3*, 135 of which are common (minor allele frequency greater than or equal to 0.05). Within this subset, three polymorphisms (rs2764264, rs13217795, and rs2802292) were associated with healthy aging and longevity [16]. The findings were independently confirmed in numerous studies [17].

Genetic association studies are also useful in identifying causal polymorphisms associated with the risk of disease. Recently, a novel association between the *FOXO3* single-nucleotide polymorphism rs13217795 and asthma susceptibility was described [18]. This finding has yet to be independently verified. To the best of our knowledge, no studies to date have investigated the association between rs13217795, a cytosine-to-thymine substitution, and allergic rhinitis.

The aim of the present study is to assess the association of rs13217795 with asthma and allergic rhinitis.

## Methods

### Participants

In this case–case–control, the initial study population consisted of 110 controls, 160 asthmatics, and 126 allergic rhinitis patients, for a total of 396 participants. The study population described herein is the aggregate of two previously described samples [19, 20]. Study participants were consecutively recruited from the Jordan University Hospital and the Jordan Hospital during the time period between August 2010 and January 2013. Asthma diagnoses (made by Professor Nathir M. Obeidat) were based on history, physical examination, and pulmonary function tests. Similarly, allergic rhinitis diagnoses (made by Professor Tareq M. Mahafza) were based on history and physical examination. In the latter cases, the skin prick test was used as an ancillary diagnostic tool. Patients with both conditions were excluded from the study. The control group comprised patients’ companions. Controls were unrelated to patients and had no history of respiratory disease, as determined by clinical interview and examination. The chief inclusion criterion for all groups was ethnicity. Participants were exclusively drawn from the same ethnic group (namely Arabs) in order to minimize population stratification. Ethnicity was determined by self-report. Institutional Review Board (Jordan University Hospital, Amman, Jordan) approval was obtained, and all participants or their guardians provided written informed consent.

### DNA extraction and genotyping

A sample of 5 mL venous blood was collected in a Vacutainer blood collection tube containing EDTA. Genomic DNA was extracted using the Wizard® Genomic DNA Purification Kit (Promega Corporation, USA). Purified DNA was stored at −20 °C, pending analysis. *FOXO3* (rs13217795) genotyping was conducted using the PCR–RFLP method. The procedure was adapted from a previous study [18]. Investigators who performed the genotyping were blinded to the participants’ case–control status. A subset of participants was not genotyped due to low DNA yield; these individuals were excluded from all subsequent analyses.

### Power analysis

A priori power analysis was conducted using the PGA software package [21]. The following assumptions were made for sample size calculations: 50% minor allele frequency, 10% disease prevalence, complete linkage disequilibrium, 5:4 case–control ratio, 5% Type I error rate, 20% Type II error rate, and one effective degree of freedom. Under the general model of disease penetrance, the anticipated odds ratios of heterozygotes and derived allele homozygotes in reference to ancestral allele homozygotes were 2.5 and 4, respectively. An odds ratio of 3 was anticipated under both the recessive and dominant models of disease penetrance. Minimum sample size estimates for the general, recessive, and dominant models of disease penetrance were 89, 57, and 97 cases, respectively. The assumptions were informed by a previous study, published guidelines, and data from the 1000 Genomes Project [18, 22].

### Data analysis

Data were manually entered into the IBM SPSS Statistics Data Editor. All data analyses were conducted using this software package, unless otherwise indicated. Descriptive statistics were generated. The Mann–Whitney U Test was used to compare the median age of each case group with that of controls. Pearson’s chi-squared test was used to test for an association between gender and case–control status. The phi coefficient (*φ*) was used as a measure of the strength of association.

Binomial logistic regression was used to relate case–control status, the response variable, to its predictors (namely, genotype, age, and gender) under the general, recessive, and dominant models of disease penetrance [23]. The linearity assumption was assessed using the Box–Tidwell test; multicollinearity was assessed using variance inflation factors; and, studentized residuals were used to detect outliers. Genotype data of controls were tested for deviation from Hardy–Weinberg equilibrium using an exact test implemented in PLINK [24]. Allele-based associations with case–control status were tested using Pearson’s chi-squared test.

All underlying assumptions were met. A *P* value <0.05 was considered to indicate statistical significance for all but one test; deviation from Hardy–Weinberg equilibrium was assessed at a threshold of 0.001 [25]. Numerical data are presented according to the recommendations of T. J. Cole [26]. Odds ratios are presented as: odds ratio (95% confidence interval). Frequencies are presented as: absolute frequency (relative frequency).

## Results

The final study population consisted of 94 controls, 124 asthmatics, and 110 allergic rhinitis patients (see Additional file 1). The demographic characteristics of the study population—namely age and gender—are shown in Table 1. The median age (range) of controls, asthmatics, and allergic rhinitis patients was 37.5 (17 to 73) years, 47.5 (17 to 84) years, and 30 (11 to 61) years, respectively. The median age of asthmatics and allergic rhinitis patients was statistically significant different from the median age of controls (*P* = 0.005 and *P* < 0.001, respectively). The proportion of female controls, asthmatics, and allergic rhinitis patients was 47%, 73%, and 56%, respectively. There was a statistically significant, weak association between gender and case–control status in the asthma arm of the study (P < 0.001, *φ* = 0.3). No such association was found between controls and allergic rhinitis patients (*P* = 0.2).

**Table 1:** Demographic characteristics of the study population

	Control^a^	Asthma	Allergic rhinitis
Number of participants	94	124	110
Median age (range) in years	37.5 (17 to 73)	47.5 (17 to 84)	30 (11 to 61)
*P* value		0.005	<0.001
Absolute (relative) count of females	44 (47%)	90 (73%)	62 (56%)
*P* value		<0.001	0.2

^a^ The control group is the reference group for all comparisons

Genotype-based associations were investigated under the general, recessive, and dominant models of disease penetrance. The results of these analyses are presented in Table 2. The control group comprised 19 (20%) ancestral allele homozygotes (CC), 54 (57%) heterozygotes (CT), and 21 (22%) derived allele homozygotes (TT). The proportion of ancestral allele homozygotes and heterozygotes was lower in both case groups compared to the control group. By reciprocity, the proportion of derived allele homozygotes in both case groups was higher; 49 (40%) asthmatics and 47 (43%) allergic rhinitis patients were homozygous for the derived allele.

**Table 2:** Case–control genotype data analysis

	Control^a^	Asthma		Allergic rhinitis	
	*n* ^b^	*n* ^b^	OR^c^ (95% CI)	*n* ^b^	OR^c^ (95% CI)
General					
CC	19 (20%)	14 (11%)	1	15 (14%)	1
CT	54 (57%)	61 (49%)	1.46 (0.64 to 3.34)	48 (44%)	1.05 (0.46 to 2.40)
TT	21 (22%)	49 (40%)	3.42 (1.37 to 8.57)	47 (43%)	2.35 (0.96 to 5.73)
*P* value			0.01		0.046
Recessive					
CC + CT	73 (78%)	75 (60%)	1	63 (57%)	1
TT	21 (22%)	49 (40%)	2.54 (1.33 to 4.85)	47 (43%)	2.26 (1.19 to 4.30)
*P* value			0.005		0.01
Dominant					
CC	19 (20%)	14 (11%)	1	15 (14%)	1
CT + TT	75 (80%)	110 (89%)	1.96 (0.89 to 4.33)	95 (86%)	1.43 (0.65 to 3.12)
*P* value			0.1		0.4

^a^ The control group is the reference group for all comparisons

^b^ Absolute (relative) counts of participants

^c^ Odds ratios with 95% confidence intervals, adjusted for age and gender

Indeed, the general model of disease penetrance was statistically significant for both arms of the study (asthma, *P* = 0.01; allergic rhinitis, *P* = 0.046). The odds of the asthma phenotype was 1.46 (0.64 to 3.34) and 3.42 (1.37 to 8.57) times higher in heterozygotes and derived allele homozygotes, respectively, compared to ancestral allele homozygotes. The corresponding odds ratios for the allergic rhinitis phenotype were 1.05 (0.46 to 2.40) and 2.35 (0.96 to 5.73), respectively.

In addition, the recessive model of disease penetrance was statistically significant (asthma, *P* = 0.005; allergic rhinitis, P = 0.01). The odds of asthma and allergic rhinitis phenotypes given derived allele homozygosity were 2.54 (1.33 to 4.85) and 2.26 (1.19 to 4.30) times higher, respectively, in reference to the pooled count of heterozygotes and ancestral allele homozygotes. The dominant model of disease penetrance was not statistically significant (asthma, *P* = 0.1; allergic rhinitis, *P* = 0.4).

There was no deviation from Hardy–Weinberg equilibrium in controls (*P* = 0.2). Thus, allele frequencies were computed, and allele-based associations were tested. The results are presented in Table 3. The relative frequency of the ancestral allele was 49%, 36%, and 35% in controls, asthmatics, and allergic rhinitis patients, respectively. Both case–control allele-based associations were statistically significant (asthma, *P* = 0.006; allergic rhinitis, *P* = 0.006).

**Table 3:** Case–control allele data analysis

	Control^a^ *n* ^b^	Asthma *n* ^b^	Allergic rhinitis *n* ^b^
Allele			
C	92 (0.49)	89 (0.36)	78 (0.35)
T	96 (0.51)	159 (0.64)	142 (0.65)
OR^c^ (95% CI)		1.71 (1.16 to 2.52)	1.75 (1.17 to 2.60)
*P* value		0.006	0.006

^a^ The control group is the reference group for all comparisons

^b^ Absolute (relative) frequencies of alleles

^c^ Odds ratios with 95% confidence intervals

## Discussion

In this study, we explored the association between an intronic single-nucleotide variant in *FOXO3* (rs13217795) and two disease phenotypes—asthma and allergic rhinitis. The two independent case groups were compared with ethnically matched controls in age- and gender-adjusted analyses. We found a significant association between rs13217795 and both diseases under multiple standard models of disease penetrance.

For both arms of the study, general model analytics suggest that more than one specific model—namely the recessive and multiplicative models—is highly plausible. Indeed, both the recessive and multiplicative models were statistically significant. The dominant model of disease penetrance did not provide significant evidence for association in either study arm.

The single-nucleotide variant under study (rs13217795) was polymorphic in our study population. The minor allele in all study groups was the ancestral allele, with a frequency of 0.49 in controls. Coincidentally, the minor (ancestral) allele frequency reported by the 1000 Genomes Project, based on 26 populations, was 0.49. Frequency data across these populations were highly variable. However, South Asian population data at the rs13217795 locus were a very close fit to the control data in this study.

The association between rs13217795 and asthma has only been explored in one previous study [18]. Incidentally, the study population in question comprised Indians of South Asia. The reported odds of the asthma phenotype were 5.54 (2.48 to 12.62) and 21.45 (8.78 to 53.84) higher in heterozygotes and derived allele homozygotes compared to ancestral allele homozygotes. The independent verification of this association in the present study, albeit to a modest extent, is of marked importance in a landscape of studies wherein the reproducibility of results is a matter of great concern [27].

The case–case–control design implemented in this study enabled a direct comparison of asthma and allergic rhinitis in terms of the risk conferred by rs13217795. Curiously, the genotypic and allelic distributions of the two diseases were strikingly similar. In turn, the significance pattern of all associations tested were identical for both case–control analyses. Odds ratios for specific alleles influencing complex diseases are expected to be in the range of 1.1 to 3.0 [28]. The odds ratios reported herein—for both case–control comparisons—fall within this range.

Aberrant expression of *FOXO3* is associated with various pulmonary pathologies [10–15]. In addition, the gene product is a transcription factor involved in immune homeostasis [9]. Thus, the association of *FOXO3* (rs13217795) with asthma and allergic rhinitis is highly plausible from a biologic standpoint. The robustness of this finding remains to be established.

Therapeutic interventions targeting the *FOXO3* gene or its products are of great interest, particularly in relation to longevity. In a recent study of healthy rats, long-term consumption of epigallocatechin gallate was associated with increased lifespan through a FOXO3-driven mechanism [29]. Additionally, curcumin has been shown to increase FOXO3-mediated gene expression by twofold [30]. Following further biologic elucidation of the role of *FOXO3* in asthma and allergic rhinitis, these phytochemicals may represent attractive therapeutic alternatives.

The main limitation of the present study is the likely causal irrelevance of rs13217795. While intronic single-nucleotide variants may very well influence gene expression, a significant association is more likely to be indirect. In that case, the marker locus may be in linkage disequilibrium with the causal variant. Indeed, rs13217795 has not been detected as an expression quantitative trait locus of *FOXO3* by the Genotype-Tissue Expression (GTEx) project [31]. Therefore, future genetic association studies of asthma and allergic rhinitis should include marker loci with putative causal relevance. In addition, anticipated odds ratios may have been overestimated in the power analysis. Therefore, the study was underpowered to detect low odds ratios despite a larger operative sample size than computed estimates. However, anticipated odds ratios were reasonably overestimated based on the results of a previous study [18]. Ultimately, the small operative sample size does not appear to compromise the study’s conclusions. The null hypothesis was rejected for both case–control comparisons under the general, recessive, and multiplicative models of disease penetrance. The pattern of significance is in support of the apparent differential distribution of genotype frequencies between cases and controls.

## Conclusions

We present the first independent verification of the association between rs13217795 and asthma. In addition, we report, for the first time, an association between rs13217795 and allergic rhinitis. By extension, this is a novel characterization of rs13217795 as a shared genetic marker for both asthma and allergic rhinitis. Marker selection in future genetic association studies of asthma and allergic rhinitis should include functional polymorphisms in linkage disequilibrium with rs13217795.

## Additional file

Additional file 1: Raw data. Demographic and genotype data for all participants. (XLS 56 kb)

## Abbreviations

COPD: Chronic obstructive pulmonary disease
*FOXO3*: Forkhead box O3
FOXO3: Forkhead box protein O3
PCR–RFLP: Polymerase chain reaction–restriction fragment length polymorphism
